# A Quick and Efficient Non-Targeted Screening Test for Saffron Authentication: Application of Chemometrics to Gas-Chromatographic Data

**DOI:** 10.3390/molecules24142602

**Published:** 2019-07-17

**Authors:** Pietro Morozzi, Alessandro Zappi, Fernando Gottardi, Marcello Locatelli, Dora Melucci

**Affiliations:** 1 Department of Chemistry “G. Ciamician”, University of Bologna, 40126 Bologna, Italy; 2 COOP ITALIA Soc. Cooperativa, Casalecchio di Reno, 40033 Bologna, Italy; 3 Department of Pharmacy, University “G. D’Annunzio” of Chieti-Pescara, 66100 Chieti, Italy

**Keywords:** saffron, adulteration, food authenticity, gas-chromatography, chemometrics

## Abstract

Saffron is one of the most adulterated food products all over the world because of its high market prize. Therefore, a non-targeted approach based on the combination of headspace flash gas-chromatography with flame ionization detection (HS-GC-FID) and chemometrics was tested and evaluated to check adulteration of this spice with two of the principal plant-derived adulterants: turmeric (*Curcuma longa* L.) and marigold (*Calendula officinalis* L.). Chemometric models were carried out through both linear discriminant analysis (LDA) and partial least squares discriminant analysis (PLS-DA) from the gas-chromatographic data. These models were also validated by cross validation (CV) and external validation, which were performed by testing both models on pure spices and artificial mixtures capable of simulating adulterations of saffron with the two adulterants examined. These models gave back satisfactory results. Indeed, both models showed functional internal and external prediction ability. The achieved results point out that the method based on a combination of chemometrics with gas-chromatography may provide a rapid and low-cost screening method for the authentication of saffron.

## 1. Introduction

The commercial product named “Saffron Powder” is a powdered spice obtained by crushing the filaments of the *Crocus sativus* L. flower [1]. Unfortunately, because of its high market price, this spice is one of the most often adulterated food products worldwide [2]. There are different kinds of possible frauds, the most frequent being the addition of foreign matter, such as derivatives from flowers of other plants, to increase the mass of the final product without adding costly pure saffron. In some cases, even total substitution of saffron powder with adulterants may be found [3].

The high market price of saffron is due to the laborious process required to obtain the spice and the limited areas of production [4]. The flower of *Crocus sativus* L. is indeed cultivated only in some regions of Asia (Kashmir, northern Iran) and Europe (Castilla la Mancha, Spain; Kozani, Greece; Abruzzo and Sardinia, Italy) [5]. Several Protected Designations of Origin (PDOs) have been created to protect the authenticity of saffron (as it has, for example, in the Italian “Zafferano dell’Aquila”, one of the major areas in terms of production and global exports) [5]. Galvin-King et al. [6] report that the business volume concerning all herbs and spices is around four billion US dollars; economists soon expect growth up to 50%. As a consequence, the business volume of frauds is estimated to cause economic damage to the global food industry in the order of several tens of billions of US dollars [7].

In order to ensure the authenticity and the quality of saffron, a standard method is proposed by the International Organization for Standardization (ISO). In particular, the last international standard regulation regarding saffron quality (ISO 3632-1:2011) [1] mainly provides a UV-Vis spectrophotometric analysis to conventionally quantify the flavor strength (expressed as concentration of picrocrocin), the aroma strength (concentration of safranal), and the coloring strength (concentration of crocin) of saffron samples. However, this method has sometimes proved incapable of evaluating saffron adulteration [8] related to spectral interferences and to the impossibility to resolve chemicals present in the adulterants that show a similar UV-Vis absorbance.

Consequently, many different analytical methods have been developed to overcome this limitation; a complete and exhaustive description of all the relevant analytical techniques is given by Kiani et al. [9]. In particular, many other spectroscopic techniques [10,11,12,13], chromatographic techniques [14,15,16], and molecular-biological techniques [17,18,19] have been exploited. Among the molecular-biological techniques, the genome-based approach, usually based on DNA extraction [20], amplification, and sequencing, represents the principal strategy to ensure the food authenticity.

However, many of these procedures are time consuming and expensive, as they require highly specialized personnel and are based on destructive methodologies.

With the aim of by-passing the above-listed drawbacks, a preliminary study for a rapid, simple, and cheap screening test for the assessment of adulterated saffron is herein developed. In particular, a non-targeted approach is used.

The non-targeted approaches are increasingly used in the field of food authenticity because they allow the examining of food fingerprints, which were previously acquired by the use of spectroscopic, spectrometric, or chromatographic techniques. This check is performed holistically and without long, complicated, and problematic identification and quantification of specific and characteristic metabolites [21].

In this work, gas-chromatographic profiles are used as chemical fingerprints, because the patterns of the most volatile compounds are characteristic for odorous spices (such as saffron and their plant adulterants) and, consequently, they may represent important variables for the assessment of saffron authenticity [22,23,24].

In particular, this study presents a combined application of Heracles II (AlphaMos, Toulouse, France) instrumentation, a headspace flash gas-chromatography with flame ionization detection (HS-GC-FID), and chemometric techniques [25]. Heracles II provides gas-chromatographic profiles of the analyzed samples rapidly and without any chemical sample pre-treatment [25,26,27,28]. Thus, the gas-chromatographic fingerprints are subsequently submitted to chemometric modeling through a multivariate approach [29,30], allowing detection of the eventual adulteration of saffron.

The focus of this work is the evaluation of saffron adulteration by two of the most frequently used plant-derived adulterants: turmeric (*Curcuma longa* L.) and marigold (*Calendula officinalis* L.).

## 2. Results and Discussion

In this work, 61 samples of commercial spices were analyzed by Heracles II flash HS-GC-FID, which meant there were 244 objects or rows of the dataset matrices. Although several peaks were present in the obtained chromatograms, for the non-targeted approach used in this work it was not necessary to associate the identified chromatographic peaks with the corresponding volatile compounds.

**Figure 1:**
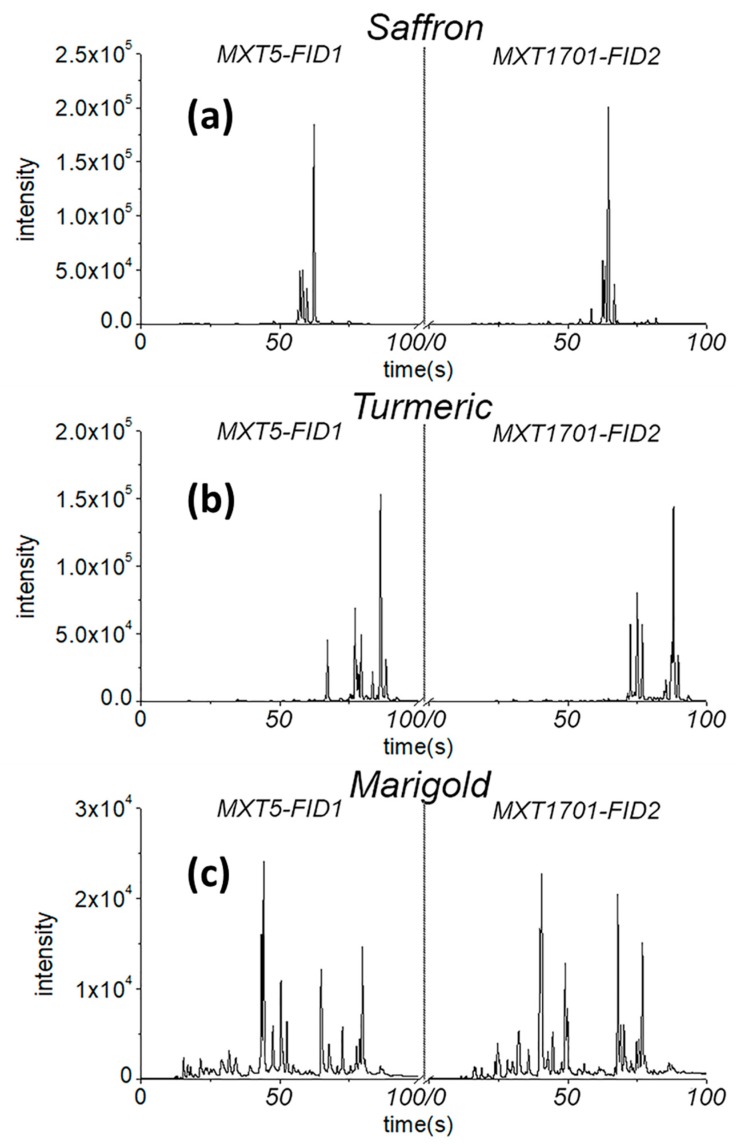
Representative gas-chromatographic (GC) fingerprints of saffron (**a**), turmeric (**b**), and marigold (**c**) obtained by Heracles II instrument. The chromatograms from column MXT5 are reported in the left part of the figure, while the chromatograms from column MXT1701 are reported on the right. These chromatograms were recorded simultaneously by the headspace flash gas-chromatography with flame ionization detection (HS-GC-FID).

Examples of the chromatograms of some analyzed samples are reported in Figure 1. It was evident that the discrimination of pure spices could be directly achieved by simply superimposing the GC chromatograms in Figure 1 without any need of chemometrics. Of course, pure samples are even distinguishable with eyes without any chemical analysis. What is interesting, however, is to discriminate *mixture* samples, which simulate adulterated saffron powders. This can be done only by chemometrics.

Even if distinguishing pure samples is trivial, it is useful to create classification models based on pure standards. In fact, the models allow quantification of the dissimilarity of mixtures with respect to pure classes through parameters that are specific for each multivariate classification method. 

From the obtained experimental data, two matrices were constructed: the area dataset (AD, 244 rows × 56 columns) and the intensity dataset (ID, 244 rows × 20,002 columns). More details will be given in the section Materials and Methods, paragraph 3.4 (“Working dataset”).

Both matrices, as described previously, were subjected to the following chemometric elaborations (LDA and PLS-DA).

### 2.1. LDA Model and Results for AD

A preliminary PCA computed on the area dataset led us to find 42 outliers—20 outliers for the “Saffron” class, eight for the “Marigold” class, and 14 for the “Turmeric” class. This brought us to a dataset with dimensions 202 (objects) × 56 (variables). On this dataset, LDA was carried out. Leave-one-out cross validation (LOO-CV) was performed to internally validate the LDA model. The results of LOO-CV, in this case, could be expressed as the percentage of well-classified samples (NER), which for this LDA model was 100%. This result was obvious, since pure samples were considered.

**Figure 2:**
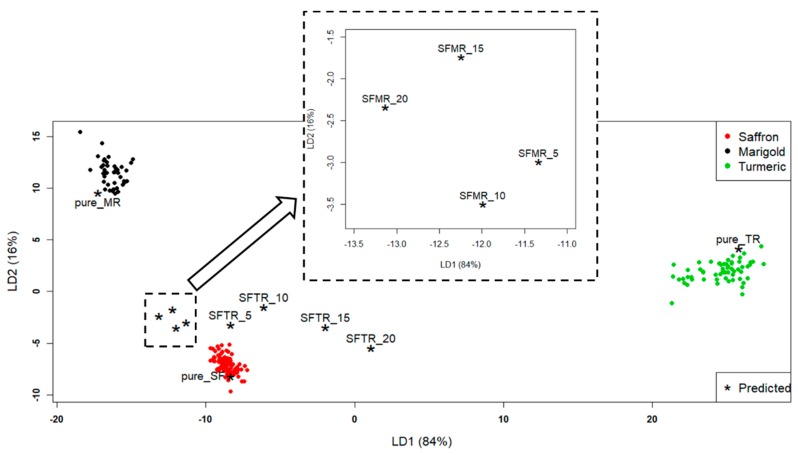
Linear discriminant analysis (LDA) discriminant plot, LD1 vs. LD2. The projected test samples (external validation results) are symbolized by asterisks (*). The graph portion inside the smaller dashed square is magnified into the greater dashed square.

The application of LDA produced the discriminant plot in Figure 2. Three clusters were evidenced, corresponding, as expected (100% NER), to the three a-priori classes (pure spices). In particular, the “Saffron” class was mostly discriminated from “Turmeric” along LD1 and from “Marigold” along LD2. Besides the three clusters, test samples were projected (asterisks). Table 1 summarizes all the test samples.

All the pure samples of the test set (pure_MR, pure_TR, and pure_SF) were assigned to the correct classes. They were correctly put inside the class spaces to which they were referred. What was particularly interesting was the behavior of the mixture samples; their distance from the pure spices clusters was significant. The mixture samples in Figure 2, although close to the “Saffron” class, moved away from it with an increasing percentage of adulterant. Moreover, the turmeric-adulterated samples (SFTR) got closer to the “Turmeric” class, moving along LD1, while the marigold-adulterated samples (SFMR) got closer to the “Marigold” class, moving along LD2. To quantify such behavior, the Euclidean distances between each point and each class centroid were computed, and the results are reported in Table 2. The class centroids were the points whose coordinates were the mean values of the coordinates of all the class objects. Thus, these could be considered as the “most representative” points for each class (although fictitious). 

From Table 2, it can be seen that the distances of the turmeric-adulterated samples (SFTR) from the “Saffron” class increased, and the distance from the “Turmeric” class decreased with an increasing percentage of adulteration. The situation was a bit more complicated for the SFMR samples, because their distances did not have a “linear” behavior with the adulterant percentage (in particular, SFMR_10 was farther from “Marigold” class than SFMR_5, and SFMR_20 was closer than SFMR_15), as can be seen from Figure 2. However, it is interesting to highlight that the distance of the farthest calibration saffron sample from the “Saffron” class centroid was 2.6. This distance could be considered as a sort of radius of the “Saffron” class, and all the mixture sample distances reported in Table 2 were higher than this value. This meant that, by computing the Euclidean distances of the projected samples from the class centroids, the LDA model could detect (at least qualitatively) a saffron sample adulterated by turmeric or marigold even down to the percentage of adulteration of 5%_w/w_.

### 2.2. PLS-DA Model and Results for ID

**Figure 3:**
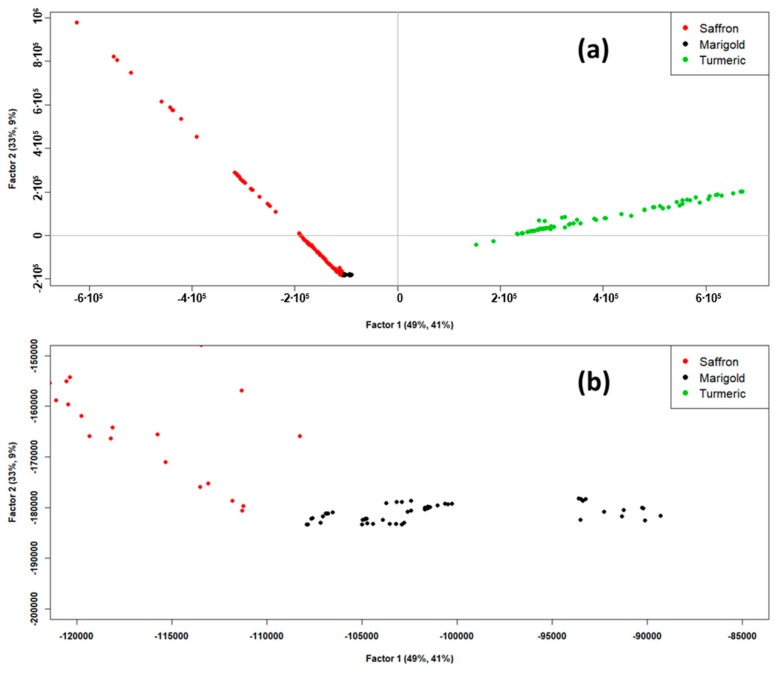
(**a**) Scores of partial least squares discriminant analysis (PLS-DA) model, Factor-1 vs. Factor-2. (**b**) Zoomed scores plot of the PLS-DA model, Factor-1 vs. Factor-2.

A preliminary PCA computed on the intensity dataset led to finding four outliers (one sample) for the “Saffron” class and five outliers for the “Turmeric” class. Moreover, to reduce the computational cost while maintaining good data representation, one variable every ten was retained [25]. In this way, the ID dataset on which PLS-DA was carried out had dimensions of 235 × 2001. PLS-DA was chosen instead of LDA for this dataset due to the high number of variables and the high co-linearity between them. LDA requires the computation of the covariance matrix of the dataset, but it is not possible when the variables are co-linear [31]. Figure 3 shows the PLS-DA scores plot. As it can be seen in Figure 3a, Factor-1 and Factor-2 of PLS-DA together explained 82% of the X-explained variance and 50% of the Y-explained variance, which could be considered satisfactory to describe the dataset. From this scores plot, good discrimination of “Saffron” and “Turmeric” classes could be observed. The “Marigold” class, on the contrary, seemed to be overlapped to the “Saffron” class in the lower left part of the scores plot (third quadrant of the plot). However, when zooming in on this overlap zone, as it can be observed in the scores plot reported in Figure 3b, these two classes were found to be resolved.

**Figure 4:**
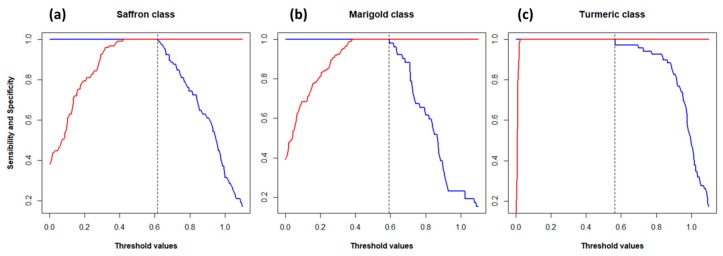
Sensitivity (blue lines) and specificity (red lines) for (**a**) “Saffron”; (**b**) “Marigold”; (**c**) “Turmeric” classes computed for each threshold value. Vertical dashed lines are the chosen thresholds for the corresponding class.

The CV was also performed to internally validate the PLS-DA model. Sensitivity and specificity for each class were computed according to Ballabio and Consonni (2013) [32] using 200 possible threshold values ranging from 0.1 to 1.1. The results are shown in Figure 4. Nine PLS-factors were used for “Saffron” and “Marigold” classes and three factors for “Turmeric” class (from Figure 3, it is easy to see that the discrimination of the “Turmeric” class was easier and required fewer factors than the discrimination of the other two). The vertical dashed lines in Figure 4 represent the chosen thresholds, which were 0.62 for “Saffron”, 0.56 for “Turmeric”, and 0.58 for “Marigold”. Thresholds were chosen as the highest value that maximized both sensitivity and specificity (1.0 or 100%) in order to have a restrictive rule for the class assignment.

At this point, the test samples reported in Table 1 were projected onto the PLS-DA model to validate it. Table 3 shows the values of the dummy variables (y_marigold, y_turmeric, and y_saffron) and their corresponding standard deviation calculated by the PLS-DA model for the test samples. The pure samples (pure_MR, pure_TR, and pure_SF) could be considered well classified. Indeed, the calculated values of the dummy variables overcame the threshold values (i.e., belonging to the class considered) related to the pertaining class of each sample, while they did not overcome the thresholds (i.e., not belonging to class considered) related to the other classes. In particular, the pure_TR sample was assigned to the “Turmeric” class with a degree of 1.0, while there was still some overlap between “Saffron” and “Marigold” classes, which made the assignment of pure_MR and pure_SF samples to the corresponding class a bit more uncertain, although still satisfactory. The classification results for the adulteration mixtures (SFMR_5, SFMR_10, SFMR_15, SFMR_20, SFTR_5, SFTR_10, SFTR_15, and SFTR_20) instead showed an interesting behavior. The threshold value of 0.62 for the “Saffron” class caused the assignment of almost all the adulterated samples to the “Saffron”, except for SFTR_15, SFTR_20, and SFMR_20, and none of the other predicted dummy values overcame the thresholds for the other classes. However, it is interesting to note from Table 3 that the degree of belonging to the “Saffron” class tended to decrease as the percentage of the adulterant increased. At the same time, the degree of belonging to the adulterant class tended to increase. Moreover, the calculated degrees of belonging to the “Saffron” class for all the mixtures were lower than the calculated degree obtained for pure_SF sample (although not significantly different for SFTR_5).

This meant that the PLS-DA model, except for some uncertainties between “Saffron” and “Marigold”, was able to discriminate the three studied spices and to detect both an adulteration with at least 15%_w/w_ of turmeric and at least of 20%_w/w_ of marigold in saffron and, at least qualitatively, some contamination in saffron with the other two spices.

### 2.3. Comparison between PLS-DA and LDA Models

PLS-DA and LDA models returned good results. Indeed, both models had good performances in LOO-CV, and both were able to determine the adulterations of saffron simulated with the test samples listed in Table 1.

In particular, PLS-DA showed some overlap and some uncertainties of classification between “Saffron” and “Marigold” classes. On the other side, the LDA model did not show any class overlap, and it was better than the PLS-DA model in the identification of the pure test samples. Both methods had good ability in the discrimination of the “Turmeric” class from the other two. However, it is important to underline that, even for pure_MR and pure_SF samples, the PLS-DA model was able to correctly classify them.

Regarding the artificial adulteration mixtures, PLS-DA and LDA had similar performances. In fact, for the mixture samples classified by the PLS-DA model, the calculated values of the dummy variables increased with the percentage of adulteration, although they never reached the thresholds, and some doubts persisted about the assignment to the “Saffron” class of such samples. However, the LDA model, by the calculation of the Euclidean distances between the test samples and the class centroids, showed some uncertainties between “Saffron” and “Marigold” classes, but it showed an excellent visual classification in the discriminant plot.

## 3. Materials and Methods

### 3.1. Samples

After an accurate commercial search, it was found that certified standards were not available (with the only exception of saffron pistils). Hence, the training-set samples were purchased in food retails; the reliability of these standards was subsequently verified through chemometric tools (see Paragraph 3.5, principal component analysis (PCA), and Hotelling). The spice samples were taken in the same period (April 2017) from several supermarkets, herbalist’s shops, and medicinal herb gardens in Emilia Romagna (Italy). It was verified that these samples arrived at the sales centers within a month before the purchase. Twenty-eight samples of saffron, 19 samples of turmeric, and 14 samples of marigold (61 total samples, “calibration samples”) were purchased by the laboratory facilities at Coop Italia. Coop Italia is one of the most important supermarket retail chains in Italy. It also has an internal food quality control laboratory in Casalecchio di Reno (Bologna, Italy), where this work was carried out.

Moreover, three samples of pure saffron, turmeric, and marigold (“test samples”) were purchased for validation purposes. The pure saffron sample was taken from a supermarket and was a product certified by the SGS certification authority with the certification “Process Control IT MI. 13.P04 STP 013/24”. Additionally, no further analyses by means of the ISO 3632-1:2011 [1] were necessary, because the commercially available samples had been controlled before their packaging and sales. The pure turmeric sample was purchased directly from a producer in the Agricultural fair of Santerno (Imola, Bologna, Italy). The pure marigold sample was taken from the Herb Garden of Casola Valsenio (Ravenna, Italy).

### 3.2. Sample Preparation

All the spice samples were stored in a dark place at low temperature until instrumental analysis. Analyses were carried out within two weeks after sample acquisition.

Regarding the calibration samples, saffron and turmeric powders did not undergo any pre-treatment, while the petals of marigold samples were powdered with Ultra Turrax Tube Drive control (IKA, Staufen im Breisgau, Germany). An aliquot of the sample was placed inside a 20-mL plastic tube with ten stainless steel spheres (5-mm diameter). The tube was subsequently sealed with the appropriate cap and was subjected to stirring at 6000 rpm for 5 min until a medium-grained powder was obtained.

Moreover, the three test samples of saffron, turmeric, and marigold (pure_SF, pure_TR, and pure_MR) were used to prepare eight artificial mixtures (SFTR_5, SFTR_10, SFTR_15, SFTR_20, SFMR_5, SFMR_10, SFMR_15, and SFMR_20) in order to simulate partial adulterations of saffron with the other spices. These samples were obtained by mixing the pure spices in different proportions to cover a wide range of adulteration degrees. In particular, four different percentages (*w/w*) of adulteration were examined: 5%, 10%, 15%, and 20%. These pure samples and mixtures did not undergo the chemometric procedure described later but were used to validate the final partial least squares discriminant analysis (PLS-DA) and linear discriminant analysis (LDA) models. 

### 3.3. Flash Gas-Chromatography (Flash-GC)

All samples from both the calibration set (training set) and the test set were analyzed according to the following procedure.

For GC analysis, an aliquot of (30 ± 3) mg of each powdered sample was placed in a 20-mL glass vial sealed with a magnetic cap. Each sample was prepared in quadruplicate to assess the repeatability and the reproducibility of the method as well as to increase the degrees of freedom of statistical problems. The replicate measurements generated four objects (rows of the dataset-matrix) for each sample. Flash HS-GC-FID analysis was performed by Heracles II instrument at Coop Italia Laboratories.

In particular, this instrument was equipped with two capillary chromatographic columns working in parallel, namely a non-polar column (MXT5: 5% diphenyl, 95% methylpolysiloxane, 10 m length, and 180 μm diameter) and a slightly polar column (MXT1701: 14% cyanopropylphenyl, 86% methylpolysiloxane, 10 m length, and 180 μm diameter) and two flame ionization detectors (FIDs) at the end of each column. GC operation, auto sampling, and chromatographic output were managed by Alphasoft V12.4 software (AlphaMos, Toulouse, France).

The parameters of the chromatographic analysis were chosen after an optimization step to avoid significant problems such as low sensitivity, overcoming of full-scale, and low peaks resolution.

The instrument was also equipped with an auto-sampler HS100 (CTC Analytics AG, Zwingen, Switzerland), which managed up to 96 samples in the same program. The sample vials were placed in a shaker oven at 50 °C and 500 rpm for 20 min. Then, the auto-sampler syringe took 5000 μL of the head-space (by piercing the silicone septum of the vial plug). The sample was injected at 100 μL s^−1^ (the injector temperature was 200 °C). The carrier gas was molecular hydrogen (H_2_) produced by an Alliance High Purity Hydrogen generator (F-dgsi, Évry, France). A solid adsorbing trap Tenax TA 60/80 (Tenax SPA, Verona, Italy) was placed before the chromatographic columns and was maintained at 40 °C and 60 kPa for 65 s while carrier gas was flowing and then heated at 240 °C. This allowed for absorption of the volatile molecules onto the trap and removal of excess air and moisture to concentrate the analytes. Analytes were then introduced into the GC columns by a rotatory valve. The column’s initial temperature was 40 °C, which was maintained at such a value for 2 s and then increased by 3 °C s^−1^ until reaching 270 °C, then it was kept at this value for 21 s. The total acquisition time was 100 s, and the signal was digitalized every 0.01 s. While a sample was injected, other samples were shaken; the entire process was automated and managed by the instrument in the absence of personnel. As a result, if 96 samples were analyzed in the same program, the overall time needed was not 20 × 96 min but about 180 min.

### 3.4. Working Datasets

After flash GC analysis, the gas-chromatographic data obtained were tabled into a source matrix (dataset). The dataset rows represented the replicates of the 61 samples (244 rows or objects, 4 replicates for each sample). The labels of the dataset columns corresponded to GC variables, which were the acquisition times derived from the digitalization of the GC signal. Each dataset cell reported the FID signal registered at the corresponding GC time for the relevant object. A further column was the class variable reporting the *a priori* class to which the relevant object belonged. Objects were grouped into classes based on their labeled identity (saffron, turmeric, and marigold).

In particular, two different datasets were created: the “area dataset” and the “intensity dataset”. The “area dataset” (AD) variables corresponded to peak areas (56 columns); these variables corresponded to the chromatographic peaks identified by the automatic integration tool of AlphaSoft. The “intensity dataset” (ID) variables were the full chromatograms recorded by Heracles II (20002 columns); cell values were the electric current intensities of FIDs. The signal was digitalized every 0.01s for 100 s (10,001 signals), and the chromatogram of the second column was appended to the one of the first column.

Both datasets were obtained from the chromatograms elaborated by Alphasoft V12.4 software.

### 3.5. Chemometrics

Before applying any of the chemometric techniques used in this work, all the data were standardized [33]. In particular, two different scaling methods were applied to the datasets: autoscaling for the “area dataset” and centering for the “intensity dataset”.

Two models for the determination of partial or total adulteration of saffron with turmeric and marigold were created and evaluated, LDA [30] and PLS-DA [29,32]. In particular, the LDA model was computed for AD, while the PLS-DA model was computed for ID.

For each dataset, the following chemometric procedure was carried out in parallel. First, for each class, the elimination of the outliers was performed by PCA and Hotelling analysis [34] at a confidence level of 95%, as already described in a previous work [25].

Then, the refined datasets including only statistically significant samples were subsequently subjected to LDA and PLS-DA. Both chemometric models were then validated by internal cross-validation (CV) [29,30] and by projecting the eleven test samples (not used for model creation) [29]. CV is a statistical technique that allowed evaluating the prediction ability of a model (i.e., the ability to determine the values of the response variables from the predictors for the test samples). CV performed the following steps iteratively: exclude some samples (randomly selected) from the training set, build the model without the excluded samples, and classify the excluded samples with this model. During this procedure, each sample of the training set was used as a test sample at least one time. However, the results of CV were different for LDA and PLS-DA.

LDA computed a model characterized by the definition of new variables starting from the original variables (in the case of AD, chromatographic peak areas) as well as in PCA. However, LDA, unlike PCA, defined linear discriminant functions (LDs) rather than principal components (PCs) that were more effective in separating the examined classes [29]. Such a model could classify unknown samples by projecting them in the LDs space. An unknown sample was always assigned to the class for which the calculated posterior probability [35] was higher; however, the distance of objects from the classes needed to be taken into account in order to finely evaluate the degree of membership to a class. 

For LDA, the CV output was represented by the confusion matrix. In this matrix, the lines represented the “a priori” classes, and the columns represented the calculated “a posteriori” classes, to which CV reassigned the samples. The ideal situation was a diagonal matrix (i.e., the matrix in which the entries outside the main diagonal were all zero) because it was the situation in which all of the samples were correctly assigned to the corresponding “a priori” classes. Subsequently, starting from the confusion matrix, it was possible to compute the “non-error-rate” (NER) as the ratio between the objects correctly classified and the total number of objects, which represented the ability of the model to correctly recognize its objects.

PLS-DA [32] is instead a regression method in which the predictor variables (X-matrix) were the experimental ones (in the ID case, the full chromatograms), while the responses (Y-matrix) were the so-called “dummy variables”. These dummy variables were the degrees of belonging to the examined classes (in this work, saffron, turmeric, and marigold) and assumed the values for calibration objects to be 0 and 1 (where 1 represented the certainty of belonging to the considered class, while 0 represented the certainty of not belonging to the considered class). The projection of an external sample onto a PLS-DA model returned a set of values for the dummy variables that could be considered as “degrees of belonging” to each class.

CV results for a PLS-DA model were represented by the calculated values of dummy variables for each sample, which meant the predicted degree of belonging of each sample to each class. These values could be used to calculate a threshold value for each class that optimized both sensitivity and specificity for the classification. The procedure for computing such threshold values is described by Ballabio and Consonni (2013) [32]. The projected samples of the test set could then be assigned to a class if their corresponding calculated value of the dummy variable overcame the threshold.

Outliers elimination was carried out by the software The Unscrambler V10.4 (Camo, Oslo, Norway), while LDA and PLS-DA were carried out (with relative CV and projections) by the software R V3.4.3 (R Core Team, Vienna, Austria) with the packages “MASS” [31] and “pls” [35]. 

## 4. Conclusions

The achieved results illustrate that the herein proposed, non-targeted strategy based on the combined application of chemometrics with Heracles II flash HS-GC-FID may provide a rapid and low-cost screening method for the authentication of saffron.

The samples were analyzed without any preparation or after a rapid grinding operation, allowing us to avoid expensive pre-treatments and any contamination before analysis by gas-chromatography. Furthermore, once the sample is put into the auto-sampler of the instrument, this instrumental analysis is entirely automated and requires a short analysis time (overall, less than 20 min for a single sample and a couple of minutes *per* sample for 96 samples simultaneously put in the auto-sampler).

Finally, with chemometrics, it was possible to use the GC data both as they are produced by the instrument (chromatograms) and by integrating the chromatographic peaks to build classification models (PLS-DA and LDA). These models had good calibration ability, evaluated by cross-validation (CV) and, most of all, good prediction ability, evaluated by projecting external test samples that simulated adulterations of saffron with turmeric and marigold. Moreover, for adulterant additions below 33%_w/w_, the official UV-VIS spectrophotometry method was not able to detect adulteration [8]. On the contrary, Heracles II combined with chemometrics allowed us to go far below this limit; a PLS-DA model able to detect down to 15 ÷ 20%_w/w_ of adulteration was validated. Moreover, a discriminant plot obtained through LDA showed significant differences between pure samples and adulterated samples down to 5 ÷ 10%_w/w_.

Another important characteristic of the chemometric approach is that it does not require the identification of the volatile compounds to create a model able to find an adulterated saffron sample. The use of the entire chromatograms ensures that all the possible markers for turmeric or marigold adulteration are taken into account in the model construction.

## Figures and Tables

**Table 1:** The test samples used for external validation: pure spices and artificial mixtures.

Test Samples	%_W/W_ of Saffron Adulteration	Code
Pure Saffron	-	pure_SF
Pure Turmeric	-	pure_TR
Pure Marigold	-	pure_MR
saffron + turmeric	5	SFTR_5
	10	SFTR_10
	15	SFTR_15
	20	SFTR_20
saffron + marigold	5	SFMR _5
	10	SFMR _10
	15	SFMR _15
	20	SFMR _20

**Table 2:** Euclidean distances of the test samples reported in Table 1 from the three class centroids.

Sample Code	Saffron	Turmeric	Marigold
**pure_SF**	1.1	34.6	21.1
**pure_TR**	36.3	2.5	42.8
**pure_MR**	18.6	42.5	2.2
**SFTR_5**	3.8	33.4	16.7
**SFTR_10**	6.2	31.0	16.4
**SFTR_15**	7.6	27.2	20.7
**SFTR_20**	9.9	24.7	24.2
**SFMR_5**	4.8	36.3	15.3
**SFMR_10**	4.8	37.1	15.6
**SFMR_15**	6.4	37.1	13.8
**SFMR_20**	6.4	38.0	14.3

**Table 3:** External validation results (calculated Ys: degrees of belonging) of the test samples projected on the PLS-DA model. The numbers in brackets are the corresponding standard deviations.

Sample Code	y_saffron	y_turmeric	y_marigold
**pure_SF**	0.78 (0.03)	0.01 (0.02)	0.21 (0.04)
**pure_TR**	−0.1 (0.2)	1.0 (0.1)	0.1 (0.2)
**pure_MR**	0.34 (0.04)	0.03 (0.02)	0.63 (0.04)
**SFTR_5**	0.71 (0.04)	0.06 (0.02)	0.23 (0.04)
**SFTR_10**	0.66 (0.07)	0.12 (0.04)	0.22 (0.08)
**SFTR_15**	0.56 (0.06)	0.26 (0.03)	0.19 (0.06)
**SFTR_20**	0.51 (0.11)	0.32 (0.06)	0.17 (0.11)
**SFMR_5**	0.69 (0.04)	0.01 (0.02)	0.30 (0.04)
**SFMR_10**	0.65 (0.03)	0.02 (0.02)	0.33 (0.04)
**SFMR_15**	0.63 (0.04)	0.02 (0.02)	0.35 (0.04)
**SFMR_20**	0.59 (0.04)	0.02 (0.02)	0.39 (0.04)

## References

[1] International Organization for Standardization (2011). ISO 3632-1. Spices—Saffron (Crocus sativus L.).

[2] Moore J.C., Spink J., Lipp M. (2012). Development and Application of a Database of Food Ingredient Fraud and Economically Motivated Adulteration from 1980 to 2010. J. Food Sci..

[3] Nazari S.H., Keifi N. (2007). Saffron and various fraud manners in its production and trades. Acta Hortic..

[4] Johnson R. (2014). Food Fraud and “Economically Motivated Adulteration” of Food and Food Ingredients.

[5] Bosmali I., Ordoudi S.A., Tsimidou M.Z., Madesis P. (2017). Greek PDO saffron authentication studies using species specific molecular markers. Food Res. Int..

[6] Galvin-King P., Haughey S.A., Elliott C.T. (2018). Herb and spice fraud; the drivers, challenges and detection. Food Control.

[7] PwC & SSAFE (2016). Food Fraud Vulnerability Assessment. http://www.pwc.com/gx/en/services/food-supply-integrity-services/assets/pwc-food-fraud-vulnerability-assessment-and-mitigation-november.pdf.

[8] Sabatino L., Scordino M., Gargano M., Belligno A., Traulo P., Gagliano G. (2011). HPLC/PDA/ESI-MS evaluation of saffron (*Crocus sativus* L.) adulteration. Nat. Prod. Commun..

[9] Kiani S., Minaei S., Ghasemi-Varnamkhasti M. (2018). Instrumental approaches and innovative systems for saffron quality assessment. J. Food Eng..

[10] Petrakis E.A., Cagliani L.R., Polissiou M.G., Consonni R. (2015). Evaluation of saffron (*Crocus sativus* L.) adulteration with plant adulterants by1H NMR metabolite fingerprinting. Food Chem..

[11] Petrakis E.A., Polissiou M.G. (2017). Assessing saffron (*Crocus sativus* L.) adulteration with plant-derived adulterants by diffuse reflectance infrared Fourier transform spectroscopy coupled with chemometrics. Talanta.

[12] Zalacain A., Ordoudi S.A., Díaz-Plaza E.M., Carmona M., Blázquez I., Tsimidou M.Z., Alonso G.L. (2005). Near-infrared spectroscopy in saffron quality control: Determination of chemical composition and geographical origin. J. Agric. Food Chem..

[13] Ordoudi S.A., De Los Mozos Pascual M., Tsimidou M.Z. (2014). On the quality control of traded saffron by means of transmission Fourier-transform mid-infrared (FT-MIR) spectroscopy and chemometrics. Food Chem..

[14] Rubert J., Lacina O., Zachariasova M., Hajslova J. (2016). Saffron authentication based on liquid chromatography high resolution tandem mass spectrometry and multivariate data analysis. Food Chem..

[15] Nenadis N., Heenan S., Tsimidou M.Z., Van Ruth S. (2016). Applicability of PTR-MS in the quality control of saffron. Food Chem..

[16] Aliakbarzadeh G., Parastar H., Sereshti H. (2016). Classification of gas chromatographic fingerprints of saffron using partial least squares discriminant analysis together with different variable selection methods. Chemom. Intell. Lab. Syst..

[17] Torelli A., Marieschi M., Bruni R. (2014). Authentication of saffron (*Crocus sativus* L.) in different processed, retail products by means of SCAR markers. Food Control.

[18] Gismondi A., Fanali F., Martínez Labarga J.M., Caiola M.G., Canini A. (2013). *Crocus sativus* L. Genomics and different DNA barcode applications. Plant Syst. Evol..

[19] Babaei S., Talebi M., Bahar M. (2014). Developing an SCAR and ITS reliable multiplex PCR-based assay forsafflower adulterant detection in saffron samples. Food Control.

[20] Danezis G.P., Tsagkaris A.S., Camin F., Brusic V., Georgiou C.A. (2016). Food authentication: Techniques, trends & emerging approaches. TrAC Trends Anal. Chem..

[21] Esslinger S., Riedl J., Fauhl-Hassek C. (2014). Potential and limitations of non-targeted fingerprinting for authentication of food in official control. Food Res. Int..

[22] Matsushita T., Zhao J.J., Igura N., Shimoda M. (2018). Authentication of commercial spices based on the similarities between gas chromatographic fingerprints. J. Sci. Food Agric..

[23] Heidarbeigi K., Mohtasebi S.S., Foroughirad A., Ghasemi-Varnamkhasti M., Rafiee S., Rezaei K. (2015). Detection of adulteration in saffron samples using electronic nose. Int. J. Food Prop..

[24] Carmona M., Zalacain A., Salinas M.R., Alonso G.L. (2007). A new approach to saffron aroma. Crit. Rev. Food Sci. Nutr..

[25] Melucci D., Bendini A., Tesini F., Barbieri S., Zappi A., Vichi S., Conte L., Gallina Toschi T. (2016). Rapid direct analysis to discriminate geographic origin of extra virgin olive oils by flash gas chromatography electronic nose and chemometrics. Food Chem..

[26] Wiśniewska P., Śliwińska M., Namieśnik J., Wardencki W., Dymerski T. (2016). The Verification of the Usefulness of Electronic Nose Based on Ultra-Fast Gas Chromatography and Four Different Chemometric Methods for Rapid Analysis of Spirit Beverages. J. Anal. Methods Chem..

[27] Wojtasik-Kalinowska I., Guzek D., Górska-Horczyczak E., Głabska D., Brodowska M., Sun D.W., Wierzbicka A. (2016). Volatile compounds and fatty acids profile in Longissimus dorsi muscle from pigs fed with feed containing bioactive components. LWT Food Sci. Technol..

[28] Górska-Horczyczak E., Wojtasik-Kalinowska I., Guzek D., Sun D.W., Wierzbicka A. (2017). Differentiation of chill-stored and frozen pork necks using electronic nose with ultra-fast gas chromatography. J. Food Process Eng..

[29] Berrueta L.A., Alonso-Salces R.M., Héberger K. (2007). Supervised pattern recognition in food analysis. J. Chromatogr. A.

[30] Bevilacqua M., Nescatelli R., Bucci R., Magrì A.D., Magrì A.L., Marini F. (2014). Chemometric classification techniques as a tool for solving problems in analytical chemistry. J. AOAC Int..

[31] Venables W.N., Ripley B.D. (2002). Modern Applied Statistics with S.

[32] Ballabio D., Consonni V. (2013). Classification tools in chemistry. Part 1: Linear models. PLS-DA. Anal. Methods.

[33] Van den Berg R.A., Hoefsloot H.C.J., Westerhuis J.A., Smilde A.K., van der Werf M.J. (2006). Centering, scaling, and transformations: Improving the biological information content of metabolomics data. BMC Genom..

[34] Jolliffe I.T. (2002). Principal Component Analysis.

[35] Mevik B.H., Wehrens R., Liland K.H. (2015). pls: Partial Least Squares and Principal Component Regression. R package version 2.5-0. J. Stat. Softw..

